# Long-Term Peritoneal Dialysis in Hereditary Transthyretin Amyloidosis: A Case Report and Management Challenges

**DOI:** 10.7759/cureus.92037

**Published:** 2025-09-11

**Authors:** Vítor Fernandes, Núria Paulo, Roberto Silva, Ana Oliveira, Ana Beco, Manuel Pestana

**Affiliations:** 1 Nephrology, Unidade Local de Saúde de São João, Porto, PRT; 2 Pathology, Unidade Local de Saúde de São João, Porto, PRT

**Keywords:** end stage renal disease (esrd), hereditary attr amyloidosis, peripheral nervous system diseases, peritoneal dialysis (pd), transthyretin amyloidosis

## Abstract

Amyloidosis is a disease characterized by extracellular deposition of misfolded proteins, causing progressive organ damage, including end-stage kidney disease (ESKD). Patients with ESKD due to amyloidosis face poor survival rates, and evidence to guide optimal management strategies is lacking. We present the case of a 64-year-old woman with hereditary transthyretin (TTR) amyloidosis and ESKD who has been successfully treated with peritoneal dialysis (PD) for over 33 months. This report highlights the challenges encountered during follow-up, including the progression of peripheral neuropathy, and underscores the need for further investigation leading to targeted therapies to prevent disease progression and complications.

## Introduction

Amyloidosis is a disease characterized by the extracellular deposition of misfolded proteins as insoluble amyloid fibrils, leading to progressive organ damage. It can be classified as localized and systemic forms of amyloidosis. The most common forms of systemic amyloidosis arise from plasma cell dyscrasia, chronic inflammation, ageing, or inherited gene mutations [1]. Systemic amyloidosis is classified based on the fibril precursor protein, with the most common types being light chain (AL), serum amyloid A (AA), and transthyretin (TTR) amyloidosis [2].

Clinical manifestations of amyloidosis vary depending on the organs affected. Commonly involved organs include the heart, kidneys, peripheral and autonomic nervous systems, and the gastrointestinal (GI) tract. Cardiac manifestations include heart failure with preserved ejection fraction, arrhythmias, and hypotension. Renal involvement usually manifests as nonselective proteinuria, progressive renal dysfunction, or nephrotic syndrome. Amyloid neuropathy is characterized by symmetric sensorimotor polyneuropathy and autonomic dysfunction, while GI involvement often presents as bowel dysmotility and malabsorption. Recognizing this broad spectrum of clinical features is essential for early diagnosis and targeted management of systemic amyloidosis [3].

TTR amyloidosis (ATTR) results from the misfolding of TTR, a transport protein synthesized by the liver. This condition can be acquired (wild-type ATTR, associated with aging) or hereditary (caused by mutations in the *TTR *gene). The hereditary form, also referred to as ATTRv (variant), is an autosomal dominant disorder, and the Val30Met mutation is the most prevalent worldwide [4]. Although ATTRv is primarily associated with progressive peripheral and autonomic neuropathy, up to one-third of patients develop proteinuria, and approximately 10% progress to end-stage kidney disease (ESKD)[5]. Currently, disease-modifying therapies such as TTR stabilizers (tafamidis) and newer small interfering RNA molecules (patisiran, inotersen, vutrisiran) are approved for the treatment of ATTRv. However, data regarding kidney outcomes, and their safety and efficacy for improving neuropathy in patients with ESKD on renal replacement therapy (RRT) remain largely unstudied [3,4].

Patients with ESKD due to amyloidosis face poor survival rates on RRT, often due to infections and cardiovascular complications, namely hypotension and rhythm disturbances [6]. Studies have reported an overall survival of 5% at 24 months after dialysis initiation in patients with AL and AA amyloidosis and 29.5% at 3 years in ATTRv amyloidosis [7,8]. Despite these poor outcomes, there is limited evidence to guide the optimal treatment strategy for TTR amyloidosis with kidney failure. Peritoneal dialysis (PD) is a recognized modality of RRT in patients with ESKD; however, reports on its use in patients with systemic amyloidosis remain scarce, especially with a long follow-up. This lack of data underscores the importance of the present case, which illustrates the feasibility and long-term outcomes of PD in hereditary transthyretin amyloidosis. Hence, we present a case of ATTRv amyloidosis and ESKD, which was successfully managed with PD for 33 months.

## Case presentation

We describe the case of a 64-year-old woman who was previously followed in the neurology department for lower limb sensory alterations, paresthesia, and gait disturbance. Electromyography demonstrated a pattern of bilateral axonal sensorimotor polyneuropathy. She also had a history of heart failure with reduced ejection fraction, left bundle branch block, and atrial flutter. In 2017, she was referred to our nephrology department for evaluation of nephrotic syndrome and acute kidney injury. Urinalysis was bland. Serum electrophoresis, serum and urine immunofixation, and serum free light chain (FLC) ratio were within normal limits. Echocardiography revealed mild left atrial enlargement, a left ventricular ejection fraction of 40%, concentric left ventricular hypertrophy, and an interventricular septal thickness of 12 mm.

**Figure 1:**
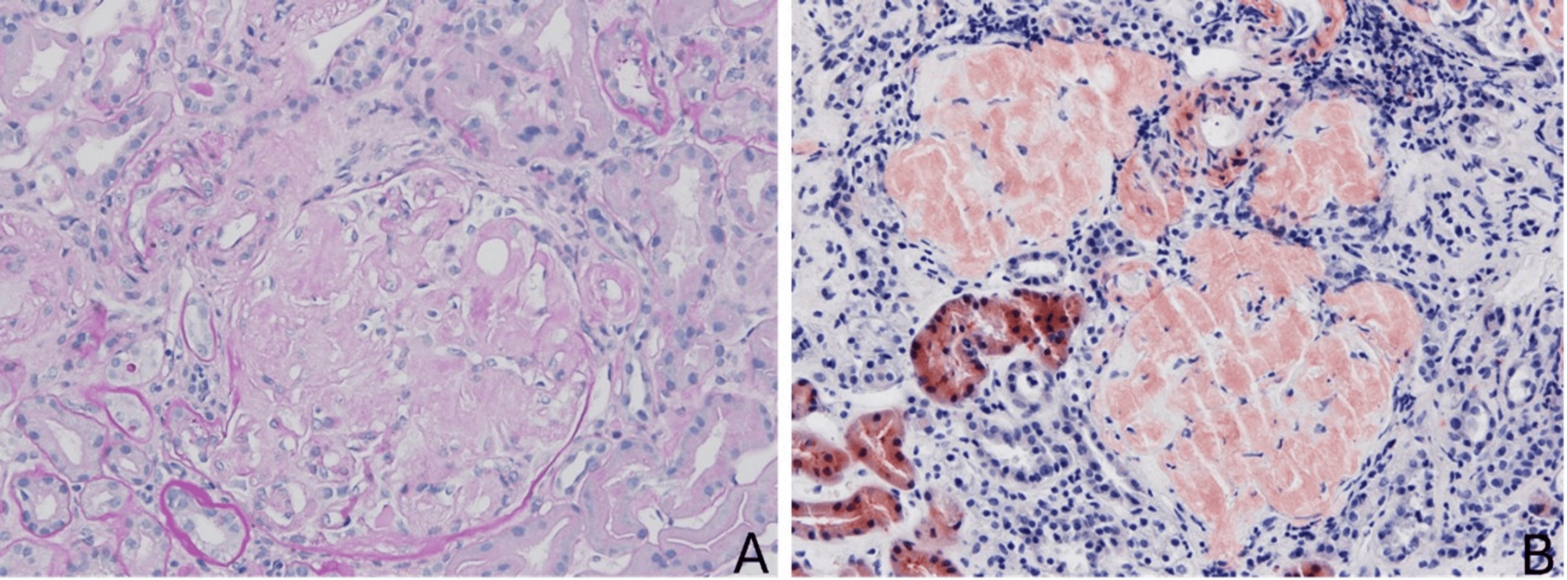
Renal Biopsy Histopathology Glomeruli showing mesangial expansion and capillary wall thickening due to amorphous eosinophilic deposits, weakly positive on Periodic Acid–Schiff (PAS) stain (Figure 1a - PAS 400X). Congo Red staining demonstrates positivity with characteristic apple green birefringence under polarized light, consistent with amyloid deposition (Figure 1b - Congo Red stain 400x).

A kidney biopsy revealed glomerular and vascular amyloid deposits, confirmed by characteristic apple green birefringence with Congo Red staining. Immunofluorescence was negative for immunoglobulins, complement, and fibrinogen (Figure 1). Genetic testing identified a heterozygous *TTR *gene mutation (p.Val50Met). A diagnosis of ATTRv amyloidosis was established based on histological and genetic findings. The remaining work-up is summarized in Table 1.

**Table 1:** Laboratory parameters during initial nephrotic syndrome evaluation. Abbreviations: ESR, erythrocyte sedimentation rate; RBC, red blood cell; IgG, immunoglobulin G; IgA, immunoglobulin A; IgM, immunoglobulin M; FLC, free light chain; SAA, serum amyloid A; C3, complement component 3; C4, complement component 4; ANA, antinuclear antibodies; dsDNA, double-stranded deoxyribonucleic acid; GPL, IgG phospholipid units; MPL, IgM phospholipid units; SGU, standard G units for β2-glycoprotein 1 IgG; SMU, standard M units for β2-glycoprotein 1 IgM; HBsAg, hepatitis B surface antigen; HCV Ab, hepatitis C virus antibody; HIV, human immunodeficiency virus.

Parameter	Value	Reference Range
Hemoglobin (g/dL)	11.3	12.0-16.0
ESR (mm/1h)	49	0-30
Serum Albumin (g/L)	30.4	38.0-51.0
Serum Creatinine (mg/dL)	1.34	0.51-0.90
Serum urea (mg/dL)	98	10-50
24-hour Urine Protein (g/24h)	4.99	<0.15g
Urine RBC (/uL)	3	<20
Serum IgG (mg/dL)	945	600-1560
Serum IgA (mg/dL)	293	50-373
Serum IgM (mg/dL)	90	40-325
Serum FLC Kappa (mg/dL)	290	200-440
Serum FLC Lambda (mg/dL)	132	110-240
FLC Kappa/Lambda Ratio	2.20	0.26-1.65
SAA (mg/L)	9.7	0.0-6.4
C3 (C3c Fraction) (mg/dL)	107	83-177
C4 (C4 Fraction) (mg/dL)	21	12-36
ANA (Antinuclear Antibodies)	1:100 (nucleolar)	<1:100
Anti-dsDNA (IU/mL)	<10.0	<100.0
Anti-cardiolipin IgG (GPL)	2.6	<20.0
Anti-cardiolipin IgM (MPL)	2.8	<20.0
β2-Glycoprotein 1 IgG (SGU)	1.9	<20.0
β2-Glycoprotein 1 IgM (SMU)	3.0	<20.0
Rheumatoid Factor (IU/mL)	<10.0	<30.0
HBsAg	Negative	–
HCV Ab	Negative	–
HIV	Negative	–

The patient was treated with tafamidis. However, her kidney function continued to deteriorate, and she began PD at our center in March 2022, based on her personal preference. At that time, tafamidis was discontinued by the neurology team due to insufficient evidence supporting its use in patients with ESKD.

Over 33 months of follow-up on PD, the patient remained clinically stable and well-adapted to the dialysis regimen. Currently, she is being treated with continuous ambulatory peritoneal dialysis (CAPD), consisting of four daily exchanges: three daytime exchanges with 2 L of 1.36% glucose solution and one overnight exchange with 2 L of icodextrin, resulting in a mean daily ultrafiltration volume of 1.0 to 1.5 L. She maintained good blood pressure control without any antihypertensive drugs and a stable nutritional status, without any hospitalizations due to hypervolemia.

Dialysis adequacy parameters included a total weekly Kt/V of approximately 2.4 and a weekly creatinine clearance of around 70 L, indicating effective metabolic control (Table 2). Regarding peritoneal solute transfer rate (PSTR), follow-up testing showed an increase in the plasma-to-dialysate (D/P) creatinine ratio from 0.74 to 0.90. This change has not had a significant clinical impact on fluid management to date.

**Table 2:** Laboratory parameters at the last follow-up. Abbreviations: iPTH, intact parathyroid hormone; NT-proBNP, N-terminal pro-B-type natriuretic peptide.

Parameter	Value	Reference Range
Hemoglobin (g/dL)	14.1	12.0-16.0
Ferritin (mg/mL)	245	20-250
Transferrin saturation (%)	42	20-50
Serum Albumin (g/L)	31	38.0-51.0
Potassium (mEq/L)	3.6	3.5-5.1
Calcium (mg/dL)	9.2	8.2-10.4
Phosphorus (mg/dL)	4.1	2.7-4.5
iPTH (pg/mL)	511	136-612
Bicarbonate (mmoL/L)	25.8	-
NT-proBNP (pg/mL)	3482	<125

During this period, the patient experienced only one episode of catheter exit site infection and one episode of peritonitis. She was hospitalized once for syncope secondary to atrioventricular block, which required implantation of a cardiac resynchronization therapy (CRT) pacemaker. Her most recent echocardiogram showed a recovered left ventricular ejection fraction, likely reflecting improved ventricular synchrony.

Despite overall stability on PD, progression of her peripheral neuropathy was noted. She developed increasing gait instability, balance impairment, and a higher risk of falls, ultimately leading to a loss of autonomy and a significant decline in her quality of life.

## Discussion

There are limited reports of patients with systemic amyloidosis treated with PD, most of which involve cases secondary to plasma cell dyscrasia or AA amyloidosis [9,10]. Given that amyloid proteins can deposit throughout the body, including potentially the peritoneal membrane, this may result in increased membrane stiffness, altered vascular permeability, and impaired solute transport efficiency. Nonetheless, peritoneal amyloid deposition remains rare and is not routinely confirmed in clinical practice [11]. In our patient, an increase in peritoneal solute transfer rate (PSTR) was observed during follow-up, transitioning to a fast transporter profile. While this may result from exposure to PD solutions over time, we cannot exclude the possibility of amyloid deposition in the peritoneal membrane, as no biopsy was performed. Importantly, higher PTSR has been associated with increased risks of mortality and hospitalization in PD patients [12].

PD may offer potential benefits compared to hemodialysis, including reduced hemodynamic instability and the removal of circulating amyloid precursors, as reported in a case of AL amyloidosis by Stone et al [13]. In our case, the patient maintained a stable hemodynamic status throughout follow-up, with no hospitalizations due to heart failure.

The patient also experienced a low rate of infectious complications over the nearly 3 years of follow-up. This is consistent with findings by Browning et al., who reported that the incidence of peritonitis in a patient with amyloidosis treated with PD was comparable to that of other patients in their unit [9].

Reports addressing long-term survival in patients with amyloidosis on PD are scarce. Grzebalska et al. described a case in which a patient remained on PD for 87 months without major complications, suggesting that PD may be a safe and feasible long-term treatment option that contributes to improved survival [10]. Similarly, our patient experienced no major complications and maintained adequate dialysis parameters over a 33-month period.

Despite these considerations, there is minimal evidence specifically evaluating outcomes in patients with ATTRv amyloidosis and ESKD treated with PD, as most available data is based on patients treated with hemodialysis. To our knowledge, only one recent case has reported the use of PD in a patient with ATTRv receiving tafamidis. In that case, a reduced dose of tafamidis was successfully administered, with the patient maintaining effective PD, stable residual renal function, and no new episodes of heart failure decompensation. However, the impact of PD and tafamidis on peripheral neuropathy in ATTRv remains unclear, as neuropathy-related outcomes were not addressed in that report [14].

Our experience corroborates the findings of the previous cases, as our patient has been successfully treated with PD for 33 months, without major infectious or cardiovascular complications, namely decompensated heart failure due to hypervolemia. Despite PD presenting as a safe and feasible option for managing ESKD complications in this patient, the interruption of a targeted therapy with tafamidis by the neurology team, since its use has not been specifically studied in a population with ESKD, could impact the progression of neuropathy. In fact, her neuropathy gradually worsened over time, significantly impacting her autonomy and quality of life. Given that tafamidis has been previously associated with no significant side effects, we should consider its use in these patients and evaluate neuropathy-related outcomes.

This report has inherent limitations, as it describes the experience of a single patient and therefore cannot be generalized. Nonetheless, it contributes to the limited literature on PD in amyloidosis-related ESKD and may encourage further studies to clarify outcomes in this setting.

## Conclusions

We believe that this case of a patient successfully treated with PD over an extended follow-up period should encourage nephrologists to consider PD as a viable option for managing ESKD in individuals with ATTRv. While PD proved to be safe and effective in maintaining dialysis adequacy and hemodynamic stability, the absence of targeted therapy was associated with worsening neuropathy, which negatively impacted the patient’s quality of life and functional status. This highlights the need for studies on the safety and effectiveness of disease-modifying treatments, such as tafamidis and RNA interference therapeutics, in patients with ESKD undergoing RRT, particularly PD. Further research is essential to evaluate whether these therapies can improve symptom control and overall clinical outcomes in this challenging patient population.
